# The Complexities of Interspecies Somatic Cell Nuclear Transfer: From Biological and Molecular Insights to Future Perspectives

**DOI:** 10.3390/ijms26073310

**Published:** 2025-04-02

**Authors:** Peachanika Pankammoon, Marvin Bryan Segundo Salinas, Chatchote Thitaram, Anucha Sathanawongs

**Affiliations:** 1 Faculty of Veterinary Medicine, Chiang Mai University, Chiang Mai 50100, Thailand; phitchanika_p@cmu.ac.th (P.P.); chatchote.thitaram@cmu.ac.th (C.T.); 2 Department of Basic Veterinary Sciences, College of Veterinary Science and Medicine, Central Luzon State University, Science City of Muñoz 3120, Nueva Ecija, Philippines; salinasmarvinbryan@clsu.edu.ph; 3 Elephant, Wildlife and Companion Animals Research Group, Chiang Mai University, Chiang Mai 50100, Thailand

**Keywords:** interspecies somatic cell nuclear transfer, interspecies cloning, embryonic development, transcriptomic analysis, genome incompatibility, nucleocytoplasmic, mitonuclear, epigenetic reprogramming, mitochondrial genomes, wildlife conservation

## Abstract

For nearly three decades, interspecies somatic cell nuclear transfer (iSCNT) has been explored as a potential tool for cloning, regenerative medicine, and wildlife conservation. However, developmental inefficiencies remain a major challenge, largely due to persistent barriers in nucleocytoplasmic transport, mitonuclear communication, and epigenome crosstalk. This review synthesized peer-reviewed English articles from PubMed, Web of Science, and Scopus, spanning nearly three decades, using relevant keywords to explore the molecular mechanisms underlying iSCNT inefficiencies and potential improvement strategies. We highlight recent findings deepening the understanding of interspecies barriers in iSCNT, emphasizing their interconnected complexities, including the following: (1) nucleocytoplasmic incompatibility may disrupt nuclear pore complex (NPC) assembly and maturation, impairing the nuclear transport of essential transcription factors (TFs), embryonic genome activation (EGA), and nuclear reprogramming; (2) mitonuclear incompatibility could lead to nuclear and mitochondrial DNA (nDNA-mtDNA) mismatches, affecting electron transport chain (ETC) assembly, oxidative phosphorylation, and energy metabolism; (3) these interrelated incompatibilities can further influence epigenetic regulation, potentially leading to incomplete epigenetic reprogramming in iSCNT embryos. Addressing these challenges requires a multifaceted, species-specific approach that balances multiple incompatibilities rather than isolating a single factor. Gaining insight into the molecular interactions between the donor nucleus and recipient cytoplast, coupled with optimizing strategies tailored to specific pairings, could significantly enhance iSCNT efficiency, ultimately transforming experimental breakthroughs into real-world applications in reproductive biotechnology, regenerative medicine, and species conservation.

## 1. Introduction

Interspecies somatic cell nuclear transfer (iSCNT) represents a promising alternative approach to somatic cell nuclear transfer (SCNT), with transformative applications in conservation, therapeutic cloning, and regenerative medicine [1,2,3]. The iSCNT technique reconstructs embryos by fusing a donor nucleus from one species with an enucleated recipient oocyte from another species, which is firstly employed to resolve challenges of sudden animal death, endangered species conservation, and extinction risks [4,5], especially critical for wildlife, given the limited availability of oocytes [6,7,8,9,10,11,12,13].

Previously, iSCNT has demonstrated success in producing full-term offspring, particularly when the donor and recipient species share a close taxonomic relationship. Notable examples include African wildcat (*Felis silvestris lybica*)–domestic cat iSCNT kittens [14], gray wolf (*Canis lupus*)–domestic dog iSCNT pups [15], a Bactrian camel (*Camelus bactrianus*)–dromedary camel (*Camelus dromedarius*) iSCNT calf [6], and a Gaur (*Bos gaurus*)–bovine (*Bos taurus*) iSCNT calf [16]. However, species-specific factors and machinery cause severe incompatibility in iSCNT embryos, resulting in high rates of embryonic developmental arrest and in vivo production failure [17,18]. This could be explained by the evolutionary divergence between donor and recipient species, based on molecular clock studies [19], as further supported by a recent meta-analysis, indicating a significant decrease in iSCNT blastocyst development with widening phylogenetic distance between donor and recipient species [20]. The phylogenetic relationship significantly affects iSCNT embryo development, particularly in inter-order pairings [8,21,22], exacerbating challenges such as incomplete epigenetic reprogramming and mitochondrial incompatibility [23].

**Figure 1:**
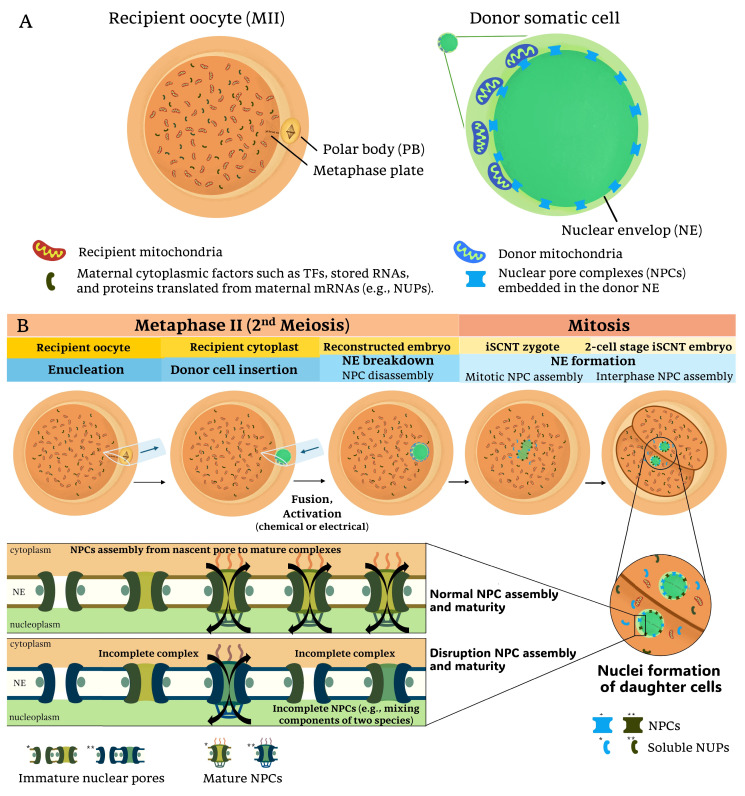
Nucleocytoplasmic interactions and nuclear pore complex (NPC) assembly in iSCNT embryos: (**A**) In interspecies somatic cell nuclear transfer (iSCNT), the mature recipient oocyte (MII stage) provides cytoplasmic factors, including mitochondria and maternally inherited proteins such as transcription factors (TFs) and nucleoporins (NUPs). The donor somatic cell, derived from a different species, contains its nuclear genome and mitochondria. The interaction between these distinct cellular components could influence the reprogramming efficiency of the reconstructed embryo. (**B**) The iSCNT process involves the enucleation of the recipient oocyte, followed by the insertion of a donor cell from a distinct species and subsequent fusion and activation. During nuclear envelope (NE) breakdown and reformation, NUPs from both recipient and donor may contribute to NPC formation in daughter cell nuclei following mitotic division. However, due to species-specific differences in NPC composition and assembly, iSCNT embryos may experience incomplete NPC maturation, leading to impaired nucleocytoplasmic communication in iSCNT embryos. Single (*) and double (**) asterisks denote color differences distinguishing donor- and recipient-derived factors, respectively.

**Figure 2:**
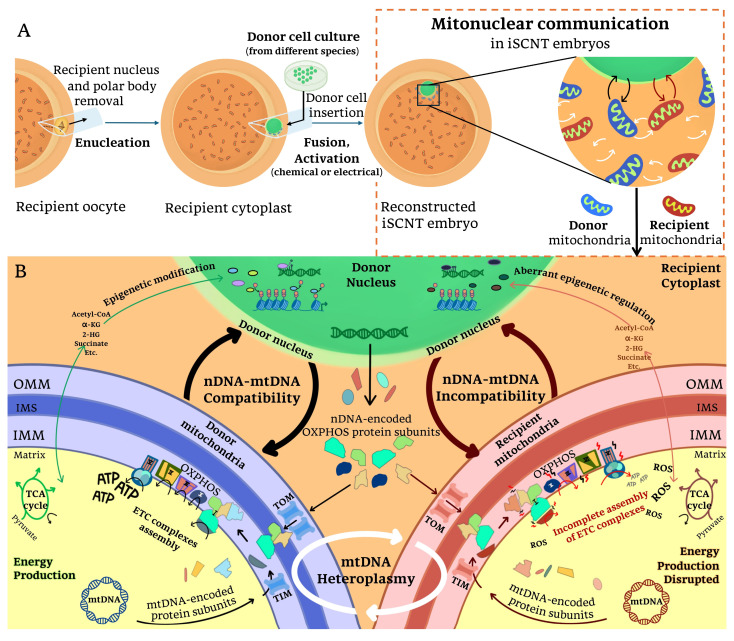
Mitonuclear incompatibility in iSCNT embryos: (**A**) Following iSCNT, mitonuclear communication occurs between donor nuclear DNA, donor-derived mitochondria DNA, and recipient-derived mitochondria DNA within the reconstructed embryo. This interaction is essential for coordinating mitochondrial function and nuclear-encoded mitochondrial gene expression. (**B**) When the nuclear genome (nDNA) and mitochondrial genomes (mtDNA) are compatible, proper mitochondrial biogenesis, oxidative phosphorylation (OXPHOS), and metabolite availability are maintained, supporting embryonic development. However, in cases of nDNA-mtDNA incompatibility, mismatches between donor nDNA-encoded and recipient mtDNA-encoded OXPHOS subunits may disrupt electron transport chain (ETC) assembly and ATP production. Impaired translocase of the outer membrane/translocase of the inner membrane (TOM/TIM) complex function, along with disruptions in tricarboxylic acid (TCA) cycle metabolite availability, could further compromise mitochondrial activity. Additionally, mtDNA heteroplasmy (the coexistence of mitochondria from different origins) may contribute to these dysfunctions by altering the cellular metabolism, interfering with epigenetic regulation, and increasing oxidative stress in iSCNT embryos. α-KG: α-ketoglutarate, 2-HG: 2-hydroxyglutarate, IMM: inner mitochondrial membrane, IMS: intermembrane space, OMM: outer mitochondrial membrane, ROS: reactive oxygen species.

Enhancing iSCNT efficiency remains limited by several unknown molecular and biological barriers. Molecular barriers include genetic issues such as genome instability and cell cycle irregularities, along with aberrant epigenetic regulations such as incomplete nuclear reprogramming and residual somatic cell memory [24,25]. Recent advancements in next-generation sequencing (NGS) technology offer significant potential for iSCNT studies by enabling the detailed exploration of transcriptomic profiles [26]. Specifically, low-input RNA sequencing (RNA-seq) facilitates the investigation of donor–recipient interactions in iSCNT embryos [26]. These interactions reveal interspecies incompatibilities that contribute to the interconnected complexities observed in iSCNT embryos. In this review, we summarize findings that highlight disruptions in nucleocytoplasmic interactions (transport between the nucleus and cytoplasm, illustrated in Figure 1), mitonuclear interactions (communication between the nucleus and mitochondria, illustrated in Figure 2), and the interplay of cytoplasm, mitochondria, and nuclear epigenome crosstalk, all of which present significant obstacles to iSCNT production [17,18,26,27,28,29].

These challenges underscore the multifaceted nature of achieving successful iSCNT outcomes. This review focuses on the hypothesis that the underlying challenges of iSCNT development are significantly influenced by nucleocytoplasmic interactions, mitonuclear communications, and mitochondrial–nuclear epigenome crosstalk. To explore this, we conducted a narrative review that summarized and synthesized the available literature on the underlying mechanisms contributing to the complexity of iSCNT and proposed potential strategies to improve iSCNT efficiency.

Our search strategy involved querying databases including PubMed, Web of Science, and Scopus using keywords such as “interspecies somatic cell nuclear transfer”, “embryonic genome activation”, “zygotic genome activation”, “nuclear reprogramming”, “epigenetic reprogramming”, “epigenetic crosstalk”, “nucleocytoplasmic interactions”, “nuclear-cytoplasmic incompatibility”, “nuclear pore complex”, “mitochondria”, “oxidative phosphorylation”, “electron transport chain”, “mitonuclear interactions”, “mitonuclear communication”, “mitochondrial genome”, “mtDNA heteroplasmy”, “nDNA-mtDNA incompatibility”, “transcriptomic analysis”, “next-generation sequencing”, “RNA sequencing”, “genome editing”, “mitochondrial base-editing”, and “embryonic development.” Studies were included if they focused on embryo and mammalian cell development with potentially relevance to iSCNT, and only peer-reviewed articles published in English were considered. No restrictions were placed on publication type. The thematic selection of studies was based on their relevance to the research question and their contribution to understanding the barriers and potential strategies in iSCNT, from almost three decades of historical research records to recent findings.

## 2. Interspecies Somatic Cell Nuclear Transfer (iSCNT) and Early Embryonic Development

iSCNT and SCNT technologies aim to reprogram somatic donor nuclei into a totipotent state, mimicking the developmental processes of a fertilized zygote [30]. The key processes in embryonic development include nuclear reprogramming and epigenetic reprogramming during the maternal-to-zygotic transition (MZT) and embryonic genome activation (EGA), to transition from maternal to embryonic control [31,32]. This process of MZT/EGA involves maternal transcription degradation, chromatin remodeling, and epigenetic modification, erasing transcriptional memory or somatic-specific patterns to allow for the activation of embryonic transcription [33,34,35,36]. Nuclear reprogramming involves structural and functional changes, including nuclear membrane breakdown, premature chromosome condensation, nuclear expansion, DNA replication, and the MZT, culminating in EGA [30,37].

The success of nuclear reprogramming in cloned embryos critically depends on the compatibility and proper signaling between the donor nucleus and recipient cytoplasm [17,27,37,38]. For example, the establishment of an open chromatin state differs between species, which facilitates EGA and epigenetic reprogramming, making the timing of mammalian EGA species-specific [39]. This species specificity introduces additional challenges in iSCNT, where the developmental timelines of the donor nucleus and recipient cytoplasm must synchronize [27,38]. Nuclear reprogramming via the iSCNT technique relies on maternal factors from recipient oocytes [40]. Mammalian oocytes possess the ability for cellular reprogramming and stemness, which is essential for animal cloning [41]. As an intrinsic factor, the recipient oocyte provides maternal cytoplasmic factors, such as transcription factors (TFs), proteins, and RNAs (Figure 1A), which need to align with the donor genome to ensure proper chromosomal DNA replication and EGA initiation during reprogramming [27,36,38,41,42,43,44].

However, iSCNT embryos frequently exhibit developmental abnormalities, leading to arrested development, which can manifest at various stages from in vitro to in vivo development [8]. The underlying molecular mechanisms of iSCNT embryo development remain discussed with particular focus on mitochondrial genome incompatibility and incomplete epigenetic reprogramming [17,18,27,31,42,45,46,47]. Instead of individual barriers accumulating to iSCNT failure, we propose that each underlying mechanism has interconnected relationships that contribute to the complexity in iSCNT production. Following a background on iSCNT and early embryonic development, we examine the key challenges and explain the complex interplay between the nucleus, cytoplasm, and mitochondria in terms of nucleocytoplasmic transportation, mitonuclear communication, and mitochondrial–nuclear epigenome crosstalk. Subsequently, we explore potential strategies and future directions, culminating in a discussion of the broader implications for iSCNT research.

## 3. Key Challenges in iSCNT Development

### 3.1. Nucleocytoplasmic Transportation in iSCNT

Following fusion and activation of reconstructed oocytes in iSCNT, nucleocytoplasmic communication initiates with nuclear envelope breakdown. During this process, nuclear pore complexes (NPCs) disassemble, redistributing soluble forms of nucleoporins (NUPs) into nucleoplasm and cytoplasm. Subsequently, the nuclear envelope reassembles, integrating newly formed NPCs into the reforming nuclear envelope of daughter cell nuclei [30,46,48,49] (Figure 1B).

#### 3.1.1. Nucleocytoplasmic Incompatibility and Nuclear Pore Complex (NPC) Formation in iSCNT

NPC architecture develops through a maturation process, starting with initial assembly at nascent pores and progressing to fully formed mature structures, involving the recruitment of numerous NUPs to build the inner and outer NPC rings [50,51,52,53,54,55]. The maturation of NPCs, composed of NUP proteins, establishes functional channels that facilitate the nucleocytoplasmic transport of maternal TFs and RNAs required for EGA [56].

In human cells, NPC assembly occurs through two main pathways: postmitotic and interphase [57]. Postmitotic assembly occurs as the nuclear envelope reassembles after mitosis, while interphase assembly allows for the insertion of new NPCs into an intact nuclear envelope during nuclear growth [57]. Both processes require coordinated interactions between NUPs and cytoplasmic scaffolding proteins to maintain proper nuclear transport. Applying this context to the iSCNT, NPC assembly should occur after the second meiotic division and nuclear envelope re-formation, subsequent to mitotic entry in the reconstructed zygote (Figure 1B). However, the functional integrity of the NPC in iSCNT embryos may be compromised due to species-specific adaptation and nucleocytoplasmic incompatibility between the donor nuclear genome and recipient cytoplasm [26,42,52,58].

While NUP genes are transcribed from the nucleus [59,60], NPC formation also depends on cytoplasmic factors, including membrane-associated proteins, chaperones, and post-translational modification enzymes [61,62]. These cytoplasmic elements regulate nucleoporin assembly, NPC maturation, and nucleocytoplasmic transport efficiency. Therefore, species-specific differences in these factors could also impair proper NPC formation, leading to defects in the nuclear import of essential TFs. This incompatibility is particularly pronounced in iSCNT embryos derived from evolutionarily distant species, as seen in murine–pig iSCNT embryos, where defective NPC formation led to failed nuclear division and disorganized NPC structures [27]. Similarly, Asian elephant–pig (AE-P) iSCNT embryos exhibited upregulation of several NPC-related genes in arrested embryos, including *NUP54*, *NUP43*, *NUP37*, *NDC1*, and *LOC126060805* (*RanBP2*) [26]. This gene upregulation may represent a compensatory response to disrupted NPC assembly, as these genes encode proteins which are critical for NPC structure and function. The NUP54, NUP43, and NUP37 proteins contribute to NPC scaffold formation, while NDC1 protein is essential for anchoring the NPC to the nuclear envelope [52]. Disruptions in *NUP37* gene impair TFs via the YAP1-TEAD pathway [48], whereas *NUP54* gene dysfunction is associated with DNA repair defects and genome instability [63]. Moreover, the Ran GTPase system, regulated by factors such as RanBP proteins, is critical for importing essential TFs and proteins [64,65,66]. In zebrafish embryos, proper NPC assembly and progressive maturation are required for efficient nuclear transport of maternal TFs into the nucleus, which regulates the onset of EGA [56,67]. A similar mechanism may occur in mammalian embryos, where NUP disruption through miRNA alterations could delay or impair the nuclear transport of pluripotency-associated TFs such as OCT4, SOX2, and NANOG [68,69]. Therefore, the observed upregulation of NPC-related genes in arrested iSCNT embryos [26] may suggest that disruptions in nuclear import could impact EGA and pluripotency maintenance. Reports of low expression of pluripotency-related genes (*OCT4*, *SOX2*, *NANOG*) in iSCNT embryos [27,58,70] further support this hypothesis.

Altogether, since NPC assembly requires both nuclear and cytoplasmic factors, the species-specific incompatibility between the donor nucleus and recipient cytoplast may lead to defective NPC formation, impaired nuclear transport, and reduced nuclear reprogramming efficiency, ultimately contributing to developmental arrest. Future research should focus on modifying cytoplasmic environments or supplementing nuclear transport regulators to improve nuclear reprogramming efficiency in iSCNT embryos.

#### 3.1.2. Nucleolar Formation and Ribosome Biogenesis in iSCNT

Nucleologenesis and ribosome biogenesis are tightly coordinated processes that begin and continue throughout the early cleavage stages of embryonic development [71]. In monkey–bovine iSCNT embryos, dysfunctional nucleoli were observed, associated with defective EGA and irregular expression of nucleolar proteins, such as upstream binding factor, fibrillarin, nucleolin, and nucleophosmin [72]. Nucleolin, a conserved nucleolar protein with a clear circular-ring structure, plays a central role in ribosome biogenesis and chromatin organization and stability, making it a key indicator of nucleolar function [38,73,74,75]. In ovine–bovine iSCNT embryos, abnormal nucleolin structures and reduced transcription levels of the nucleolin gene (*C23*) were also detected [75]. Overexpression of *C23* improved blastocyst rates, restored nucleolin structure, and upregulated ribosomal subunit-related genes, while *C23* knockdown exacerbated structural abnormalities and downregulated ribosomal subunit-related gene expression [75]. These findings suggest that abnormalities in nucleolin expression and structure in iSCNT embryos can impair nucleolar function, ribosome biogenesis, and EGA, contributing to nucleocytoplasmic incompatibility and developmental arrest [72,74,75,76].

### 3.2. Mitonuclear Communication in iSCNT

Mitochondria, maternally inherited organelles, are essential for energy production and play critical roles in embryogenesis by regulating key developmental processes [35,77,78]. Mitonuclear communication refers to the intricate interplay between the mitochondrial genome (mtDNA) and nuclear genome (nDNA), ensuring cellular function and adaptation [79]. This communication operates through the primary pathways, including anterograde signaling (from the nucleus to mitochondria) and retrograde signaling (from mitochondria to the nucleus), as well as mitonuclear feedback signaling and proteostasis regulation, allowing cells to maintain homeostasis and respond to mitochondrial stress [79]. Compatibility between nDNA- and mtDNA-encoded genes is vital for ATP generation via oxidative phosphorylation (OXPHOS), the primary energy source during early embryogenesis [80,81]. OXPHOS occurs in the inner mitochondrial membrane (IMM), where electron transport chain (ETC) complexes (I-V) generate ATP [82]. While Complex II is fully encoded by nDNA, the other ETC complexes (I, III, IV, and V) require coordinated contributions from both nDNA and mtDNA for proper assembly and function [82,83,84,85].

During the fusion and activation steps of iSCNT, mitonuclear incompatibility arises when a donor nucleus is transferred into an enucleated recipient cytoplast, introducing a small amount of donor cell-derived mitochondria (Figure 2A) [28,86]. This process results in mtDNA heteroplasmy—the coexistence of donor and recipient mtDNA [28,47,86]—and a mismatch between donor nDNA and recipient mtDNA, termed nDNA-mtDNA incompatibility (Figure 2B) [17,18,26,27]. These incompatibilities impair ETC complex assembly and OXPHOS function, disrupting downstream processes such as MZT/EGA transitions, somatic cell reprogramming, cell signaling, gene regulation, and differentiation [17,18,27,45,46,81,87,88]. For instance, in rhesus monkey–bovine embryos, the low amino acid sequence homology (75%) of the mtDNA-encoded protein cytochrome b in ETC Complex III likely contributed to incomplete function and poor blastocyst formation, possibly due to insufficient ATP production [45].

Previous studies suggest that OXPHOS defects in iSCNT embryos become more apparent after EGA [27]. However, findings from AE-P iSCNT embryos indicate that these disruptions can arise as early as the two-cell stage, possibly preceding EGA [26]. Developmentally arrested embryos at this stage exhibited compromised ETC Complexes I and II, with altered expression of key OXPHOS-related nuclear genes, including *NDUFC2*, *NDUFS3*, *NDUFAB1*, *SDHC*, and *SDHB* [26]. These disruptions are likely caused by nDNA-mtDNA incompatibility, leading to incomplete ETC subunit assembly and inefficient mitochondrial regulation [26], despite mtDNA replication not occurring during early embryogenesis in most species [78,89]. Moreover, in Przewalski’s gazelle–bovine iSCNT embryos, mitonuclear incompatibility is also evident [18]. The expression of mitochondrial translocase genes related to the TOM/TIM (translocase of the outer/inner mitochondrial membrane) complex, including *TOMM40*, *TIMM10*, *TIMM50*, and *TIMM22*, was significantly lower in the iSCNT compared to their bovine SCNT counterparts [18]. The TOM complex at the outer mitochondrial membrane (OMM) facilitates the import of nDNA-encoded mitochondrial proteins into mitochondria, while the TIM complexes mediate the translocation and integration of specific proteins into the IMM [90,91]. These findings underscore how mismatches between nDNA and mtDNA can disrupt mitochondrial protein transport and OXPHOS efficiency in iSCNT embryos.

Additionally, uncontrolled mtDNA heteroplasmy can exacerbate mitochondrial dysfunction and genetic instability in iSCNT embryo development (Figure 2B). In Lycaon–dog iSCNT embryos, mtDNA heteroplasmy has been shown to impair pyruvate metabolism and energy production, contributing to developmental failure in the fetuses [47]. Additionally, the accumulation of tricarboxylic acid (TCA) cycle metabolites can further influence epigenetic modifications through mitochondrial and nuclear epigenome crosstalk [29,92]. These observations underscore the tight interdependence between nuclear and mitochondrial genomes. Even low levels of mtDNA heteroplasmy can disrupt nuclear gene transcription, particularly genes involved in ETC function and the TCA cycle [93]. Furthermore, nuclear loci play a critical role in regulating mtDNA replication and maintenance pathways, influencing mtDNA copy number and heteroplasmy. Changes in nuclear genetic factors can therefore alter mtDNA heteroplasmy dynamics [94,95]. Collectively, these findings highlight the importance of mitonuclear communication in maintaining cellular energy balance and mitochondrial function. The interplay between mtDNA–nDNA incompatibility and mtDNA heteroplasmy adds another layer of complexity to the challenges of iSCNT embryo development.

### 3.3. Interplay of Cytoplasm, Mitochondria, and Nuclear Epigenome Crosstalk in iSCNT

Nucleocytoplasmic compatibility (e.g., NPC assembly and maturation), mitonuclear interactions (e.g., nDNA-mtDNA incompatibility and mtDNA heteroplasmy), and mitochondrial–nuclear epigenome crosstalk illustrate the intricate network of barriers in iSCNT. These interrelated challenges can disrupt the MZT/EGA, impair erasure of somatic cell memory, and affect overall epigenetic reprogramming [25,26,96]. For instance, NUPs that form the mature NPC mediate macromolecular transport between the nucleus and cytoplasm, whereas some NUPs in their soluble forms within the nucleoplasm also regulate gene activity by recruiting TFs, chromatin remodelers, RNA helicases, and mRNA export complexes, potentially affecting transcription initiation [49,97]. iSCNT studies, such as bovine–pig embryos, show failure in transcription initiation during MZT/EGA due to the absence of RNA Polymerase II accumulation and nucleoli formation [31], while Przewalski’s gazelle–bovine embryos exhibit insufficient maternal RNA degradation and reduced expression of RNA polymerase genes (e.g., *POLR2B* and *POLR3A*), suggesting impaired MZT/EGA [18]. Recent low-input RNA-seq analyses of AE-P iSCNT embryos reveal partial embryonic transcription and less differentially expressed genes (DEGs) detection [26]. A small number of DEGs in iSCNT embryos may indicate a less robust EGA process in iSCNT models than those in SCNT counterparts [18,26,75,98]. Additionally, the retention of somatic memory genes, such as *FLRT2*, *ADAMTS1*, and *FOXR1* in porcine early-dividing SCNT embryos and *JUN*, *IKBKB*, *IKBKG*, and *FOSL2* along with aberrant expression of epigenetic-related genes (e.g., *DNMT1*, *KDM5B*, and *KDM4A*) in timely developing AE-P iSCNT embryos, indicates incomplete reprogramming that hinders the establishment of totipotency [26,36,96,99]. Stage-specific effects of epigenetic regulator gene expression are evident in mouse cloned embryo development [100]. Specifically, decreased *KDM4B* gene expression results in arrest at the two-cell stage, and decreased *KDM5B* gene expression results in arrest at the four-cell stage [100]. This stage-specific regulation may also be relevant to AE-P iSCNT embryos, as *KDM4A* and *KDM5B* exhibit aberrant expression in both two-cell and four-cell stages [26]. Consequently, early cleavage does not ensure developmental success, as disrupted transport of maternal transcription factors and regulatory molecules may further impede MZT/EGA and epigenetic remodeling, underscoring the critical role of nucleocytoplasmic compatibility in establishing a totipotent state [18,26,34,96,99].

Building on the interplay between nuclear, cytoplasmic, and mitochondrial compatibility in shaping EGA and epigenetic reprogramming, recent studies indicate that mitochondrial function directly influences the regulation of the nuclear epigenome [29,92]. Although epigenetic modifiers, such as histone deacetylase inhibitors (HDACi), have been applied to address epigenetic reprogramming in SCNT/iSCNT [24,40,101], their outcomes remain inconsistent across species [16,18,102,103]. For instance, trichostatin A (TSA) improved blastocyst formation in macaque–pig iSCNT embryos [103] but had no effect on gaur–bovine and human–rabbit iSCNT embryos [16,102]. Similarly, valproic acid (VPA) failed to enhance development in Przewalski’s gazelle–bovine and macaque–pig iSCNT embryos [18,103]. These inconsistent results suggest that targeting epigenetic reprogramming alone may be insufficient to overcome iSCNT challenges. Besides factors from species-specific and experimental conditions, the recent role of mitochondria in nuclear epigenome regulation reveals functions beyond energy production [29,92]. Therefore, the aberrant epigenetic reprogramming in iSCNT embryos may arise from mitochondria–nuclear interactions.

Mitochondrial metabolites generated through the TCA cycle, including acetyl-CoA, α-ketoglutarate (α-KG), and 2-hydroxyglutarate (2-HG), regulate critical roles in regulating the nuclear epigenome by influencing chromatin modifications, DNA methylation, and histone post-translational modifications (Figure 2B) [29,104]. Disruptions in these metabolites due to nDNA-mtDNA incompatibility and mtDNA heteroplasmy can lead to aberrant epigenetic reprogramming and developmental instability [29,86,92,93]. For example, mtDNA heteroplasmy reduces mitochondrial acetyl-CoA availability, impairing histone H4 acetylation, while moderate heteroplasmy elevates α-KG levels, affecting histone H3 methylation [92]. Similarly, imbalances in mitochondrial and nuclear NAD^+^/NADH ratios disrupt histone acetylation and overall transcriptional regulation [92]. Notably, restoring mitochondrial function can reverse these epigenetic alterations, potentially through nuclear compensatory evolution [92,105].

### 3.4. Potential Strategies for iSCNT Production

Ongoing technological advancements, including optimized nuclear transfer techniques, culture systems, and genome editing tools such as CRISPR-based and mitochondrial base-editing, hold potential for enhancing iSCNT efficiency and overcoming developmental limitations. Over the past decades, sequencing technologies, including NGS and multi-omics analysis, have significantly enhanced our understanding of the molecular mechanisms underlying preimplantation embryo development [39,100,106]. For example, assay for transposase-accessible chromatin using sequencing (ATAC-seq) and RNA-seq have been used to study chromatin dynamics and transcriptional regulation during preimplantation, providing species-specific insights into EGA timing [39]. These tools can similarly be applied to iSCNT embryos to uncover species-specific dynamics in embryonic control across donor–recipient pairings. Our prior study using low-input RNA seq on fluorescent labeling of transgenic AE-P iSCNT embryos identified developmental barriers, including nucleocytoplasmic incompatibility, mitonuclear incompatibility, and aberrant epigenetic reprogramming [26]. These findings highlight the importance of RNA-seq in transcriptomic analysis for improving iSCNT reliability and advancing our understanding of cellular reprogramming challenges in different donor–recipient pairings. Additionally, an essential step in iSCNT transcriptomic analysis is the accurate identification of the donor genome, as technical errors during nuclear transfer may result in remnants of the recipient genome in iSCNT embryo samples [8]. One approach to addressing this issue is using transgenic donor cells labeled with fluorescent markers to visually identify embryos containing the donor genome [26]. Alternatively, in cases where fluorescent tagging is not used, sequencing workflows can first align clean reads to the recipient genome and then map unmapped reads to the donor genome for pooled sample analysis [107]. These bioinformatic approaches help distinguish donor-derived sequences and improve the accuracy of transcriptomic and genomic assessments in iSCNT studies.

Enhancing nDNA-mtDNA compatibility and reducing mtDNA heteroplasmy represent additional challenges in iSCNT. Strategies such as depleting mtDNA from donor cells can lower heteroplasmy in bovine SCNT, whereas preserving donor mtDNA may enhance nucleocytoplasmic coordination through selective replication in iSCNT [108,109]. Similarly, chemical depletion of mtDNA in recipient oocytes using agents such as 2′,3′-dideoxycytidine has been found to improve blastocyst formation in murine–porcine iSCNT when supplemented with immature mitochondria from embryonic stem cell (ESC) extracts [27]. Another approach, cytoplasmic bisection via handmade cloning protocol, involves manually removing visible mtDNA from bovine oocytes post-centrifugation [110], although its practical application is limited in porcine oocytes due to their dark cytoplasm [111].

Beyond depletion strategies, forced mitophagy—the selective degradation of mitochondria—offers opportunity to reduce mtDNA compatibility [112]. Mitophagy was initially explored in mitochondrial replacement therapy (MRT) to replace defective mtDNA with healthy mtDNA, reducing the risk of mtDNA carryover in embryos [113,114,115,116]. Since both MRT and iSCNT share similar challenges of genome mixing, nDNA-mtDNA incompatibility, and mtDNA heteroplasmy [117], applying forced mitophagy with NGS such as whole-mtDNA sequencing, whole-genome sequencing, whole-exome sequencing, and mitoexome sequencing enables the identification of mitochondrial mutations and assessment of heteroplasmy levels [112,118,119]. These detection technologies help in optimizing mtDNA compatibility for iSCNT.

Recent studies highlight nuclear compensatory evolution as a potential mechanism for restoring mitonuclear compatibility through nuclear-encoded proteins that regulate mitochondrial biogenesis, ETC function, mtDNA abundance, and heteroplasmy [94,105,120]. For instance, targeting nuclear genes such as *Mfn1*, which regulates mitochondrial fusion, has shown potential in improving mitochondrial density, ATP production, and embryo development in ovine–bovine iSCNT models [121]. By enhancing functional coordination between mitonuclear components, earlier studies have relied on vector-based overexpression and RNAi knockdown to modulate nuclear genes, such as *Mfn1* [121]. However, recent advancements highlight CRISPR/Cas9 as a more precise and flexible tool for targeting nuclear-encoded mitochondrial genes [122,123]. Another application of this technology, Conservation Mitonuclear Replacement (CmNR), involves editing nuclear-encoded mitochondrial genes (N-mt loci) using CRISPR/Cas9 to align them with the mitochondrial donor species, followed by nuclear transfer into enucleated oocytes [117].

In addition to nuclear gene editing, alternative mtDNA-editing tools, such as mitochondrial zinc-finger nucleases (mtZFNs) [124,125], mitoTALENs [126,127], and a mitochondrial base-editing technique called double-stranded DNA-specific cytidine deaminase-derived cytosine base editors (DdCBEs) [128,129], offer promising approaches for manipulating mtDNA. However, mtDNA editing carries risks, including unintended genomic instability, such as mtDNA fragments integrating into nuclear DNA, and off-target effects that can disrupt mitonuclear crosstalk [130,131,132]. To address these challenges, strategies such as nuclear export signal tagging, co-expressing of exonuclease proteins such as TREX1 or TREX2, and combination approaches of co-injecting mitoTALENs and DdCBEs into mouse embryos have been developed to improve mitochondrial genome stability, minimize off-target effects, and enhance the precision of these tools [130,131,132].

Optimizing in vitro culture conditions is another crucial factor in improving iSCNT embryo development, since cloned embryos rely heavily on energy provided by the culture medium and recipient cytoplasmic factors [40,54]. Tailoring culture conditions to species-specific requirements may improve developmental outcomes, for example, mouse–porcine embryos cultured in species-specific media of porcine zygote medium-3 (PZM-3) supplemented with Chatot-Ziomek-Bavister (CZB) media [133], which are effective media for porcine and mouse embryo cultures, respectively. These formulations consider species-specific metabolic demands, such as pyruvate-based support for EGA in pigs and high potassium concentrations essential for mouse embryos [134,135].

Ensuring nDNA-mtDNA compatibility is essential for supporting ATP generation, reactive oxygen species (ROS) regulation, and mitochondrial biogenesis [17,27,105]. Excessive ROS production due to nDNA-mtDNA mismatch can trigger oxidative damage through a vicious cycle that impairs mitochondrial OXPHOS [136,137,138]. Antioxidant supplementation, such as vitamin C, during in vitro maturation or throughout the manipulation steps, including fusion, activation, and reconstructed embryo culture, helps to protect against mitochondrial and DNA damage caused by mechanical stress, reduces ROS level, and supports nuclear reprogramming by modulating gene expression and DNA methylation [139,140,141,142,143]. Additionally, small molecule treatments, such as coenzyme Q10, resveratrol, and alpha-lipoic acid, optimize mitochondrial activity, reduce stress, and enhance ATP production during oogenesis and embryogenesis [144,145,146,147,148,149,150,151]. While oxidative stress management may not directly enhance iSCNT efficiency, it alleviates mechanical and cellular stress during production and early embryonic development. These strategies should be adjusted to species-specific metabolic variations in donor–recipient iSCNT pairing.

## 4. iSCNT Future Perspectives

The complexity of iSCNT research points towards a future where its unique capabilities are harnessed for species conservation and regenerative medicine. Despite the outcome limitations of iSCNT, particularly regarding low blastocyst formation rates and developmental abnormalities [8], its unique potential remains one of the few feasible methods for reproductive cloning and reproducing endangered species or potentially resurrecting extinct ones [2,6,21,22]. Among comparative nuclear reprogramming approaches, iSCNT and iPSC technologies represent unique strengths and limitations [152]. While iPSCs offer valuable tools for disease modeling, regenerative medicine, and cell-based therapies [153,154], iSCNT uniquely enables the generation of viable offspring, making it crucial for reproductive cloning and species conservation efforts [1,2,6,14,15,16].

To overcome current challenges, significant progress in advanced technologies is crucial for improving iSCNT efficiency. This progress is demonstrated by the transition from traditional comparative studies to molecular-level analyses. Previously, comparative studies on recipient oocytes from different species helped determine which pairings resulted in higher blastocyst development in iSCNT embryos [38,155]. With advances in sequencing technologies, identifying species that provide more suitable recipient oocytes is now more feasible by analyzing key gene regulatory networks and molecular pathways in different iSCNT models [26,41,107,156], making the process more reliable. For instance, integrating single-cell RNA sequencing (scRNA-seq), chromatin immunoprecipitation sequencing (ChIP-seq), and ATAC-seq could reveal regulatory genes and chromatin modifications during specific embryonic stages [39,100]. Given the species-specific molecular requirements across mammalian embryonic development [40], identifying key genes and conducting functional validation studies following NGS [157,158] could further clarify iSCNT mechanisms across different donor–recipient pairings.

Building upon this foundation, as technological advancements continue, integrating NGS with multi-omics approaches will be crucial for identifying the complexities that arise during development in each iSCNT model, thereby guiding the selection of appropriate strategies to enhance iSCNT efficiency, whether through advanced imaging techniques, mitochondrial manipulation, or genome editing tools. For example, the functional integrity of the NPC in iSCNT embryos could be compromised by species-specific adaptations and nucleocytoplasmic incompatibilities [26]. To delve deeper into nucleocytoplasmic transport dynamics, advanced imaging techniques, such as fluorescence correlation spectroscopy (FCS), super-resolution microscopy, and fluorescence recovery after photobleaching (FRAP), allow researchers to map the stoichiometry and dynamics of key NPC components during early embryonic development, particularly during nuclear envelope formation [57,159,160,161,162]. These approaches could be applied to visualize NPC assembly and transport dynamics in real time in iSCNT embryos.

Shifting to considerations in mitochondrial manipulation, previous studies have highlighted the importance of carefully selecting the source and quantity of supplemented mitochondria following mtDNA depletion [27,93,110]. Improper supplementation could disrupt genomic balance within embryos by impairing the transcription of nuclear genes critical for ETC assembly and TCA cycle function [27,93]. For instance, the choice between autologous or heterologous mtDNA, and between mtDNA from somatic cells or immature mtDNA derived from ovarian stem cells, needs careful consideration [163,164].

Moreover, strategies such as the CRISPR/Cas system and mitochondrial manipulating tools have been explored in MRT to address mitochondrial mutations, mitochondrial diseases, or mtDNA carryover [112,131,165,166]. Therefore, while MRT and iSCNT share similar challenges related to mitochondrial genome mixing [117], the approaches used in MRT could potentially be adapted for iSCNT production. In addition, when considering the direct application of CRISPR/Cas9, distinctions arise between nuclear and mitochondrial genome editing. CRISPR/Cas9 is effective for editing the nuclear genome or nuclear-encoded mitochondrial genes that affect mitochondrial function [122,123]. However, exploiting CRISPR/Cas9 to mitochondrial genome editing presents significant challenges [131,167], due to the absence of repair mechanisms in mitochondria and inherent mitochondrial characteristics [168,169,170,171,172,173,174,175,176]. Specifically, mitochondria possess very limited DNA repair mechanisms compared to the nucleus, lacking effective double-strand break (DSB) repair mechanisms and primarily relying on base excision repair pathways for oxidative damage [168,169,170,171,172,173]. Furthermore, the rapid degradation of linearized mtDNA and difficulties in delivering guide RNAs into the mitochondrial matrix hinder the precision of CRISPR/Cas9 for mtDNA editing [174,175,176].

While mitochondrial manipulation and genome editing tools hold potential for addressing nDNA-mtDNA incompatibility and mtDNA heteroplasmy, they also raise concerns regarding genomic instability, off-target effects, and other side effects [112,130,131,132,177]. Furthermore, the interconnected complexities of iSCNT suggest a potential cause-and-effect relationship, where adjustments to genes or pathways related to mitochondrial function or nucleocytoplasmic transport can have cascading effects on cell cycle regulation and epigenetic reprogramming. Given that these technologies are relatively new to embryonic and iSCNT research and are still in their initial stages of development [109,110,112,166], integrated strategies must be carefully tailored to species-specific requirements to minimize complications. Moreover, long-term studies are essential to assess the potential side effects on mitochondrial function and embryo development. However, despite these challenges, exploring the efficacy of different protocols for precise targeted editing—including mtDNA depletion, supplementation, forced mitophagy, CRISPR/Cas9, or mitochondrial base-editing—is crucial for optimizing these methods. Careful management and assessment of the consequent effects on mitochondrial function and embryo development are also necessary. Ultimately, these technologies offer valuable insights for improving iSCNT efficiency. Thus, the future perspective of iSCNT revolves around the refinement of these capabilities and addressing underlying limitations to expand its broader applicability.

## 5. Conclusions

Taken together, the findings discussed highlight the interconnected complexities of nucleocytoplasmic and mitonuclear compatibility, as well as the epigenetic crosstalk between mitochondria and the nucleus in iSCNT. Given the limited success of epigenetic modifiers alone [16,18,102,103], it is plausible that the challenges in iSCNT extend beyond isolated epigenetic issues. We hypothesize that the disruption of the interplay between nucleocytoplasmic transport and mitonuclear communication leads to its own effects and a cascade of events that ultimately impair epigenetic reprogramming. For example, compromised mitochondrial function could disrupt cellular energy homeostasis, which is essential for the proper regulation and maintenance of epigenetic marks. Similarly, aberrant channels for molecular exchange through nucleocytoplasmic transport could hinder the delivery of key reprogramming factors to the nucleus. These disruptions, in turn, could result in incomplete epigenetic reprogramming, abrupt embryonic transcription initiations, cell cycle dysregulation, and developmental arrest iSCNT.

Emerging cutting-edge technologies have significantly advanced our understanding of the biological and molecular mechanisms underlying the complexities of iSCNT. This review emphasizes that the success of iSCNT models requires not addressing a single factor in isolation but rather adapting and balancing various species-specific factors on a case-by-case basis. Strategies to improve donor–recipient species pairing must be grounded in a thorough evaluation of foundational knowledge, including developmental timing and mechanisms (e.g., MZT/EGA), epigenetic reprogramming compatibility, phylogenetic relationships, mitochondrial compatibility, embryo culture conditions, and insights from previous research and success rates. These elements form the foundation for selecting and applying advanced sequencing tools, genome editing technologies, and predictive models to address species-specific challenges effectively.

The remaining challenges in iSCNT highlight the significant research efforts required to advance this technology. The integration of tools, including single-cell sequencing, gene editing, and other novel approaches, has the potential to overcome these limitations. As these technologies mature, they will accelerate progress in iSCNT research, bridging the gap between experimental breakthroughs and practical applications. Ultimately, advancements in iSCNT will drive innovations in therapeutic cloning, regenerative medicine, and species conservation, offering transformative solutions for some of the most pressing biological and ecological challenges of our time.

## Data Availability

Not applicable.

## Abbreviations

The following abbreviations are used iIMMn this manuscript:

α-KG	α-ketoglutarate
ATAC-seq	Assay for transposase-accessible chromatin using sequencing
ATP	Adenosine triphosphate
ChIP-seq	Chromatin immunoprecipitation sequencing
CmNR	Conservation mitonuclear replacement
CRISPR/Cas9	Clustered regularly interspaced short palindromic repeats/CRISPR-associated protein 9
CZB	Chatot-Ziomek-Bavister medium
DdCBEs	Double-stranded DNA-specific cytidine deaminase-derived cytosine base editors
DEGs	Differentially expressed genes
DNA	Deoxyribonucleic acid
EGA	Embryonic genome activation
ESC	Embryonic stem cell
ETC	Electron transport chain
FCS	Fluorescence correlation spectroscopy
FRAP	Fluorescence recovery after photobleaching
H3	Histone H3
H4	Histone H4
HDACi	Histone deacetylase inhibitors
2-HG	2-Hydroxyglutarate
IMM	Inner mitochondrial membrane
IMS	Intermembrane space
iSCNT	Interspecies/Inter-order somatic cell nuclear transfer
mRNA	Messenger ribonucleic acid
MRT	Mitochondrial replacement therapy
mtDNA	Mitochondrial DNA, Mitochondrial genome
mtZFNs	Mitochondrial zinc-finger nucleases
MZT	Maternal-to-zygotic transition
NAD^+^/NADH	Nicotinamide adenine dinucleotide (oxidized form/reduced form)
nDNA	Nuclear DNA, Nuclear genome
NGS	Next-generation sequencing
NPC	Nuclear pore complex
N-mt loci	Nuclear-encoded mitochondrial genes
NUP	Nucleoporin
OMM	Outer mitochondrial membrane
OXPHOS	Oxidative phosphorylation
PZM-3	Porcine zygote medium-3
RNA	Ribonucleic acid
RNA-seq	RNA sequencing
ROS	Reactive oxygen species
SCNT	Somatic cell nuclear transfer
scRNA-seq	Single-cell RNA sequencing
TCA	Tricarboxylic acid cycle
TFs	Transcription factors
TIM	Translocase of the inner mitochondrial membrane
TOM	Translocase of the outer mitochondrial membrane
TSA	Trichostatin A
VPA	Valproic acid
